# A pull-down and slot blot-based screening system for inhibitor compounds of the podoplanin-CLEC-2 interaction

**DOI:** 10.1371/journal.pone.0222331

**Published:** 2019-09-25

**Authors:** Nobuo Watanabe, Masako Kidokoro, Yusuke Suzuki, Makiko Tanaka, Shigeaki Inoue, Hideo Tsukamoto, Noriaki Hirayama, Pei-Wen Hsieh, Ching-Ping Tseng, Yoshihide Nakagawa, Sadaki Inokuchi

**Affiliations:** 1 Department of Emergency and Critical Care Medicine, Tokai University School of Medicine, Isehara, Kanagawa, Japan; 2 Department of the Education and the Research Support Center Tokai University School of Medicine, Isehara, Kanagawa, Japan; 3 Institute of Advanced Biosciences, Tokai University, Isehara, Kanagawa, Japan; 4 Graduate Institute of Natural Products, College of Medicine, Chang Gung University, Taoyuan, Taiwan, Republic of China; 5 Department of Anesthesiology, Chang Gung Memorial Hospital, Taoyuan, Taiwan, Republic of China; 6 Department of Medical Biotechnology and Laboratory Science, College of Medicine, Chang Gung University, Taoyuan, Taiwan, Republic of China; 7 Graduate Institute of Biomedical Science, College of Medicine, Chang Gung University, Taoyuan, Taiwan, Republic of China; 8 Department of Laboratory Medicine, Chang Gung Memorial Hospital, Taoyuan, Taiwan, Republic of China; Duke University School of Medicine, UNITED STATES

## Abstract

Podoplanin, a transmembrane glycoprotein, is overexpressed in certain types of tumors and induces platelet aggregation by binding to C-type lectin-like receptor 2 (CLEC-2) on the platelet membrane. Activated platelets release granule components, which in turn, trigger epithelial-mesenchymal transition and confer invasive capacity to the tumor cells. Therefore, blocking the podoplanin-CLEC-2 interaction by a small-molecule compound is a potential therapeutic strategy to prevent cancer metastasis and invasion. To effectively identify such inhibitory compounds, we have developed a pull-down-based inhibitory compound screening system. An immunoglobulin Fc domain-CLEC-2 fusion protein was used as a bait to capture podoplanin derived from podoplanin-overexpressing HeLa cells in the presence and absence of the test compound. The protein complex was then pulled down using protein A beads. To shorten the turnaround time, increase throughput, and decrease the workload for the operators, centrifugal filter units were employed to separate free and bound podoplanin, instead of using customary aspiration-centrifugation washing cycles. Slot blotting was also utilized in lieu of gel electrophoresis and electrical transfer. Thus, the use of our pull down screening system could facilitate the effective selection of potential inhibitor compounds of the podoplanin-CLEC-2 interaction for cancer therapy. Importantly, our methodology is also applicable to targeting other protein-protein interactions.

## Introduction

Podoplanin is a type I transmembrane glycoprotein expressed in certain cells, such as lymphatic endothelial cells and kidney podocytes [1,2]. Podoplanin expression is elevated in several types of tumors, in particular, squamous cell carcinoma and glioblastomas and its levels are negatively correlated with the prognosis of the patients after surgical resection [2,3]. Among the potential roles of podoplanin, platelet activation has recently gained interest as a potential key mechanism for the malignancy of podoplanin-positive tumor cells [1,2]. That is, podoplanin binds to the platelet membrane receptor, C-type lectin-like receptor 2 (CLEC-2), via its platelet aggregation-stimulating (PLAG) domains and activates platelets [4,5]. Among other platelet releasates, TGF-β has been shown to play a key role in the induction of epithelial-mesenchymal transition (EMT), which confers invasive capacity to tumor cells [6]. Thus, interference of the interaction between podoplanin in cancer cells and CLEC-2 in platelets using small-molecule compounds, is a potential therapeutic strategy to block tumor metastasis and invasion. Indeed, several neutralizing antibodies against podoplanin have been shown to have anti-metastatic activity in mouse pulmonary metastasis models [7–9]. Nevertheless, very few small-molecule inhibitors are currently available [10,11].

Protein-protein interactions have conventionally been assessed using pull-down assays, which involve repeated washing of carrier beads to separate bound and free ligands, followed by gel electrophoretic separation and western blotting for detection. However, these procedures are too time-consuming and labor-intensive to apply in large-scale drug screening assays. To effectively identify small-molecule inhibitors of the podoplanin-CLEC-2 interaction from a compound library, we have established an efficient screening system for inhibitory compounds, based on the combination of a pull-down assay using a centrifugal filter unit and a slot blot assay.

## Materials and methods

### Reagents

Protein A beads (KANEKA KanCapA^™^) were from Wako Chemicals (Osaka, Japan). X-tremeGENE HP was purchased from Sigma (St Louis, MO, USA). Sepasol was purchased from Nacalai Tesque (Kyoto, Japan). The anti-podoplanin antibody (NZ-1, rat IgG) was purchased from Angio Bio Co. (Del Mar, CA, USA) and the anti-CLEC-2 antibody (AF1718, goat IgG) was from Novus Biologicals (Littleton, CO, USA). Horseradish peroxidase (HRP)-labeled anti-human IgG Fab2 antibody (709–1317) was purchased from Rockland Immunochemicals Inc. (Limerick, PA, USA), anti-goat IgG-HRP (P0160) from Dako (Glostrup, Denmark) and anti-Rat IgG-HRP (7077S) from Cell Signaling Tech (Danvers, MA, USA). A set of plasmids for constructing a lentivirus expression vector system, including human immunodeficiency virus type 1 (HIV-1)-based CSII-MCS-Venus, pCAG-HIVgp, and pCMV-VSV-G-RSV-Rev, were obtained from RIKEN (Wako, Osaka, Japan). The Fc-fusion protein expression plasmid containing human IgG1 (pFUSEN-hG1Fc) was purchased from InVIVOGen (San Diego, CA, USA). Hematoporphyrin was from MedChemExpress (Monmouth Junction, NJ, USA). 2CP was synthesized as reported previously [10].

### Establishment of podoplanin-expressing HeLa cells

mRNA was isolated from TE11 cells using Sepasol and a cDNA library was prepared using a High-Capacity cDNA Reverse Transcription Kit (Thermo Fisher Scientific, Waltham, MA, USA), according to the respective manufacturer’s protocols. The full-length open reading frame of podoplanin (GenBank: AF390106.1) was flanked with Age1 and BamH1 sites at the 5′ and 3′ end, respectively, and was amplified by PCR (KOD Plus; Toyobo, Osaka, Japan). The DNA sequences of the PCR primers were as follows:

Podoplanin-F: 5′-ACGACCGGTATGTGGAAGGTGTCAGCTCT and
Podoplanin-R: 5′-CGTGGATCCTTAGGGCGAGTACCTTCCCG.

The PCR fragments and CSII-MCS-Venus plasmid were digested with Age1 and BamH1 and the two fragments were ligated. The plasmids were amplified in *E*. *coli* DH5α strain and purified by using Fast Gene Midi kit (NIPPON Genetics Co, Ltd, Tokyo, Japan) according to the manufacturer’s instructions. The DNA sequence for the podoplanin region of the vector was confirmed by DNA sequencing. Finally, the podoplanin-encoding CSII vector was converted into a lentivirus vector along with the accessory plasmids, pCAG-HIVgp and pCMV-VSV-G-RSV-Rev, using HEK293 cells, and generated virus was infected in HeLa cells (MOI = 15), as described previously [12]. Ten days after infection, the Venus-positive cells were then enriched by FACS (FACSAria, BD Biosciences, San Jose, CA, USA). After expansion, the podoplanin (pod)-HeLa cells were lysed with a lysis buffer (0.5% Triton X-100, 50 mM Tris HCl [pH 7.4], 150 mM NaCl, protease inhibitor cocktail [11873580001; Roche, Basel, Switzerland]), followed by centrifugation at 10,000 x g for 10 min to obtain a podoplanin-enriched cell lysate.

### Preparation of Fc-CLEC2 fusion protein

A cDNA library was prepared in a previously published study from peripheral mononuclear cells isolated from the blood of a healthy volunteer [12]. The extracellular region of CLEC-2 (351–890, NM_016509) was amplified by PCR with BamH1 and EcoRV sites at the 5′ and 3′ ends, respectively, using the following primers:

CLEC-2-F: 5′-CGTGGATCCATGCTGGGGATTTGGTCTGTCAT and
CLEC-2-R: 5′-CGTGATATCTTAAGGTAGTTGGTCCACCTTGG.

The CLEC-2 sequence was ligated to pFUSEN and amplified, as conducted for the construction of the podoplanin-CSII vector. The DNA sequence for the CLEC-2 region of the vector was confirmed by DNA sequencing. For protein expression, the plasmid was transfected into HEK293 cells, using X-tremeDNA transfection reagent (Sigma), with a DNA to reagent ratio of 2 μg:3 μL. The following day, the culture medium was replaced with Defined K-SFM (Thermo Fisher Scientific 10785) or serum-free MEM-α medium (Nacalai 21444–05) and harvested 2 days later. The pooled medium was loaded onto a HiTrap Protein A HP Column connected to an AKTA chromatography system (GE Healthcare, Chicago, IL, USA). Fc-fusion proteins were eluted with 0.58% acetic acid and immediately neutralized with sodium borate buffer (pH 8.5). The solvent was then replaced with PBS and concentrated by ultrafiltration (Amicon-Ultra-4; Millipore, Bedford, MA). Approximately 300 μg of pure fusion protein was obtained from 1 L of conditioned medium.

### Pull down of podoplanin with Fc-CLEC-2

Typically, 20 μL of Fc-CLEC-2 (25 μg/mL = 0.42 μM) in PBS was combined with 20 μL of pod-HeLa cell lysates (1.5 mg/mL) in lysis buffer and kept on ice for 2 h, followed by capture of the complex with 10 μL of protein A beads (KanCapA^™^, 50% slurry) for 30 min. The beads with the captured protein complex were then transferred to a 0.65 μm pore centrifugal filter unit (UFC30DV00 ultra-free, Millipore) and washed four times by the addition of 0.5 mL of Tris-buffered saline (TBST; 0.05% Tween 20, 150 mM NaCl, 20 mM Tris-HCl [pH 7.5]), followed by brief centrifugation. Then, 120 μL of 0.1 M GlyHCl (pH 2.8) was loaded onto the beads on the filter tube and incubated at room temperature for 10 min. Dissociated proteins were then eluted into a new collection tube by centrifugation and immediately neutralized with 6 μL of 1 M Tris-HCl (pH 9.0). Aliquot (typically, 10 μL) was diluted to 200 μL with PBS and blotted onto polyvinylidene difluoride (PVDF) membranes, using a vacuum-assisted 48-well slot blotter (MilliBlot, Millipore). The membrane was blocked with 5% skim milk in TBST and podoplanin or CLEC-2 proteins were probed with anti-podoplanin antibody (167 ng/mL) or anti-CLEC-2 antibody (67 ng/mL), respectively, followed by appropriate HRP-conjugated secondary antibody (both 3,000 fold dilution). Chemiluminescence reactions were performed using an HRP substrate (Millipore) and the signal was recorded with an image analyzer (ATTO, Tokyo, Japan). Slot intensity was quantitated using NIH Image J software. In some experiments, proteins in the acid-elution fractions were separated by SDS-PAGE using 10% polyacrylamide gels, transferred onto PVDF membranes, and visualized as above.

### Statistics

Experimental data were analyzed by ANOVA followed by the Tukey test using GraphPad PRISM 5 (GraphPad, San Diego, CA, USA).

## Results

### Properties of podoplanin and Fc-CLEC-2

**Fig 1:**
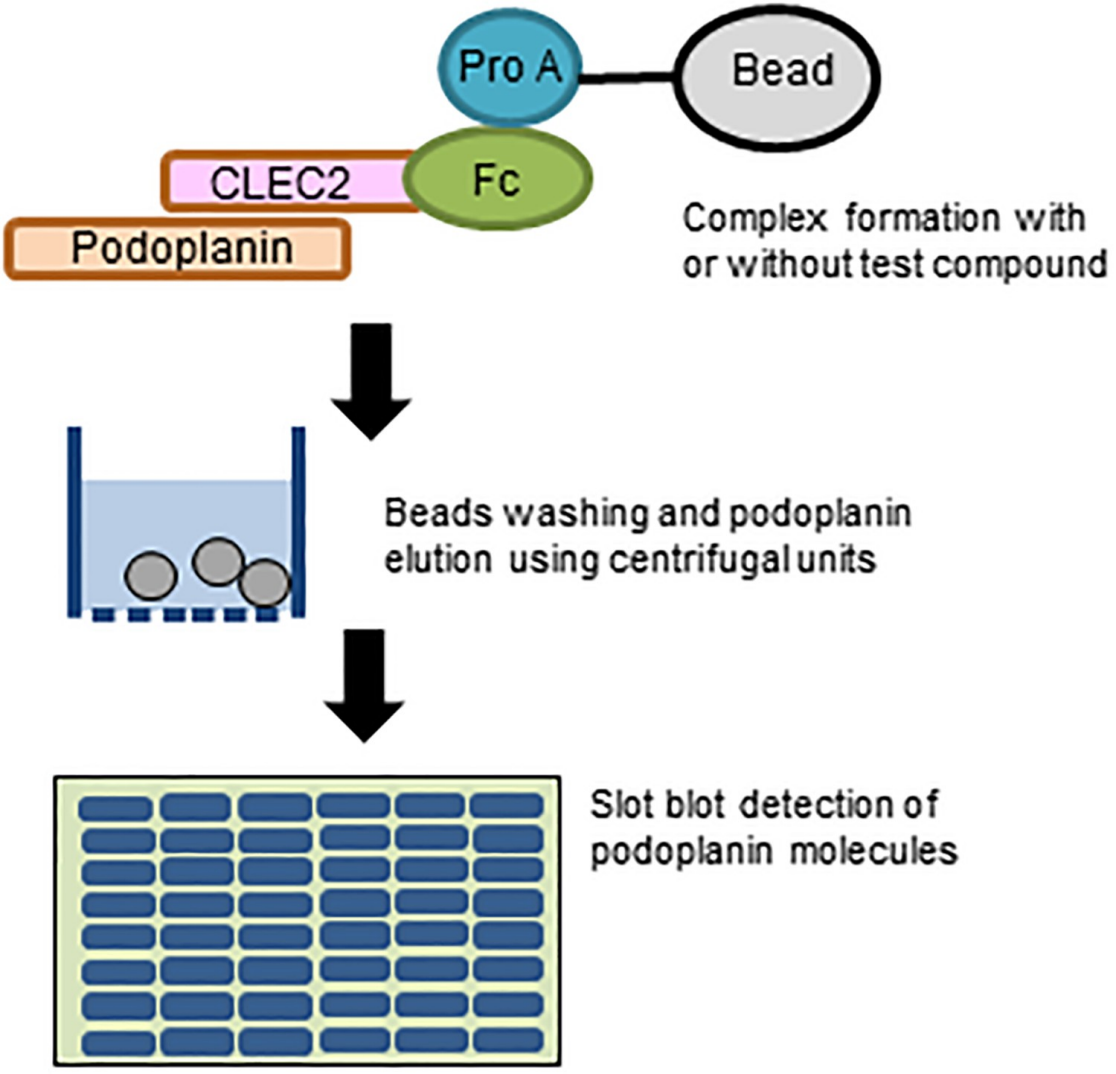
Schematic representation of podoplanin pull-down by Fc-CLEC-2 and protein A beads and subsequent slot blot analysis. Podoplanin and Fc-CLEC-2 were allowed to form complexes in solution with or without a test compound, and the protein complex was then captured by protein A-beads. The tripartite protein beads complexes were then transferred into a 0.5 mL centrifugal filter unit and centrifuged to remove unbound podoplanin and other components through the filter membrane. After four repeated washes by cycles of TBST addition and centrifugation of the tripartite protein beads complexes on the filter unit membrane, podoplanin and Fc-CLEC-2 were dissociated from the protein A-beads by acidification and eluted through the filter. The acid elution fraction was neutralized and then slot blotted onto a PVDF membrane for immunodetection of podoplanin or Fc-CLEC-2. If a test compound is capable of inhibiting the interaction between podoplanin-CLEC-2 through binding to either podoplanin or CLEC-2 moiety or both, slot intensity for podoplanin will be decreased. If a test compound inhibits the interaction between Fc moiety and protein A, slot intensity for Fc-CLEC-2 is expected to decrease.

**Fig 2:**
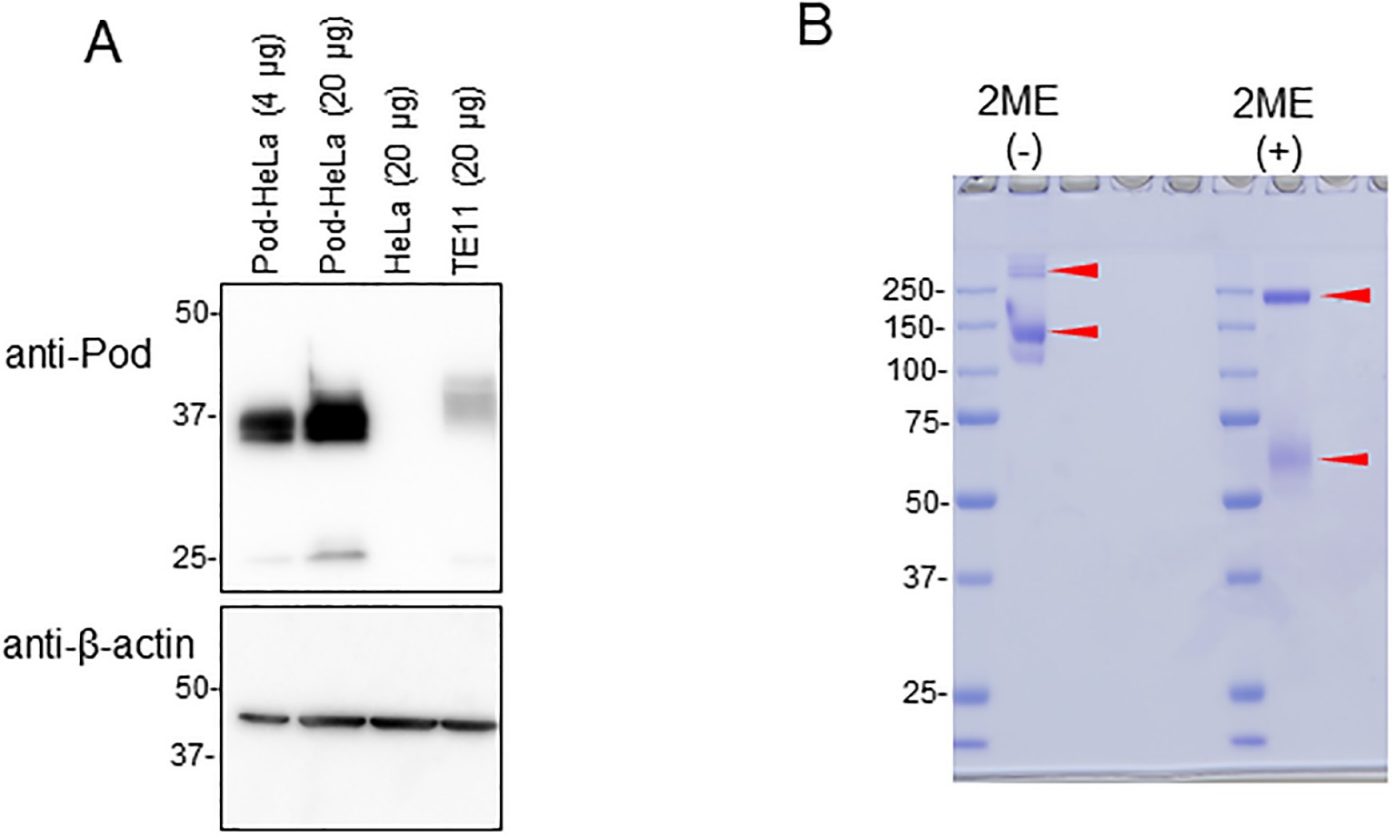
Level of podoplanin expression in HeLa cells and the purity of the Fc-CLEC-2 fusion protein. (A) Western blot analysis of podoplanin expression levels in pod-HeLa cells, parental HeLa cells, and TE11 cells. Triton X-100-soluble fractions from the indicated cells (4 μg or 20 μg) were analyzed by western blot for podoplanin expression levels. The aliquots were also analyzed for β-actin. (B) SDS-PAGE and Coomassie blue staining analysis of Fc-CLEC-2 molecules under reducing and non-reducing conditions with 2-mercaptoethanol (2ME). Red arrows indicate major bands. Data shown are a representative result from at least two independent experiments with similar results.

We planned to capture full-length podoplanin by Fc-CLEC-2, then pull down the complex using protein A-beads in a centrifugal filter unit, and detect the bound podoplanin by slot blotting (Fig 1). As a source of podoplanin molecules for the assay, as well as in the scope of future bioassays of potential hit compounds, we established a stable HeLa cell population that over-expresses podoplanin. The podoplanin expression level in these cells was more than 5-fold the expression level in esophagus carcinoma TE11 cells (Fig 2A), which are reported to express relatively high levels of podoplanin compared with other carcinoma cell lines [13].

The extracellular domain of CLEC-2 was expressed as a Fc-fusion protein in HEK293 cells and secreted into the culture medium. Although reports show that HEK293 cells endogenously express podoplanin (S1A Fig) [14], which raised concerns that a portion of Fc-CLEC-2 might be trapped by podoplanin expressed on the cell surface, we recovered a significant amount of Fc-CLEC-2 from the culture medium after transient transfection (approximately 300 μg from 1 L medium). The purity of the final product was determined, with or without 2-mercaptoethanol (2-ME) reduction, by SDS-PAGE and coomassie blue staining (Fig 2B). Although the molecular weight of the fusion protein was expected to be 60 kDa, most of the proteins appeared around 130 kDa, with some minor species > 250 kDa under non-reducing conditions. After 2-ME reduction, monomeric 60 kDa species were observed. However, significant amount of ~200 kDa species still remained after 2-ME reduction. CLEC-2 molecules have four intra-molecular disulfide bonds and form non-disulfide-linked homodimers [15]. Expression of Fc moiety alone in this cell line exclusively produced a disulfide-bonded dimeric form of ~60 kDa, but it was completely reduced by 2-ME to a monomeric form of 30 kDa (S1B Fig). It may be possible that the presence of the CLEC-2 domain somehow results in the stabilization of intermolecular disulfide bonds between the Fc moieties, which renders them 2-ME-resistant, as observed for other intermolecular disulfide-bonded proteins [16].

### Optimum conditions for podoplanin and Fc-CLEC-2 interaction

**Fig 3:**
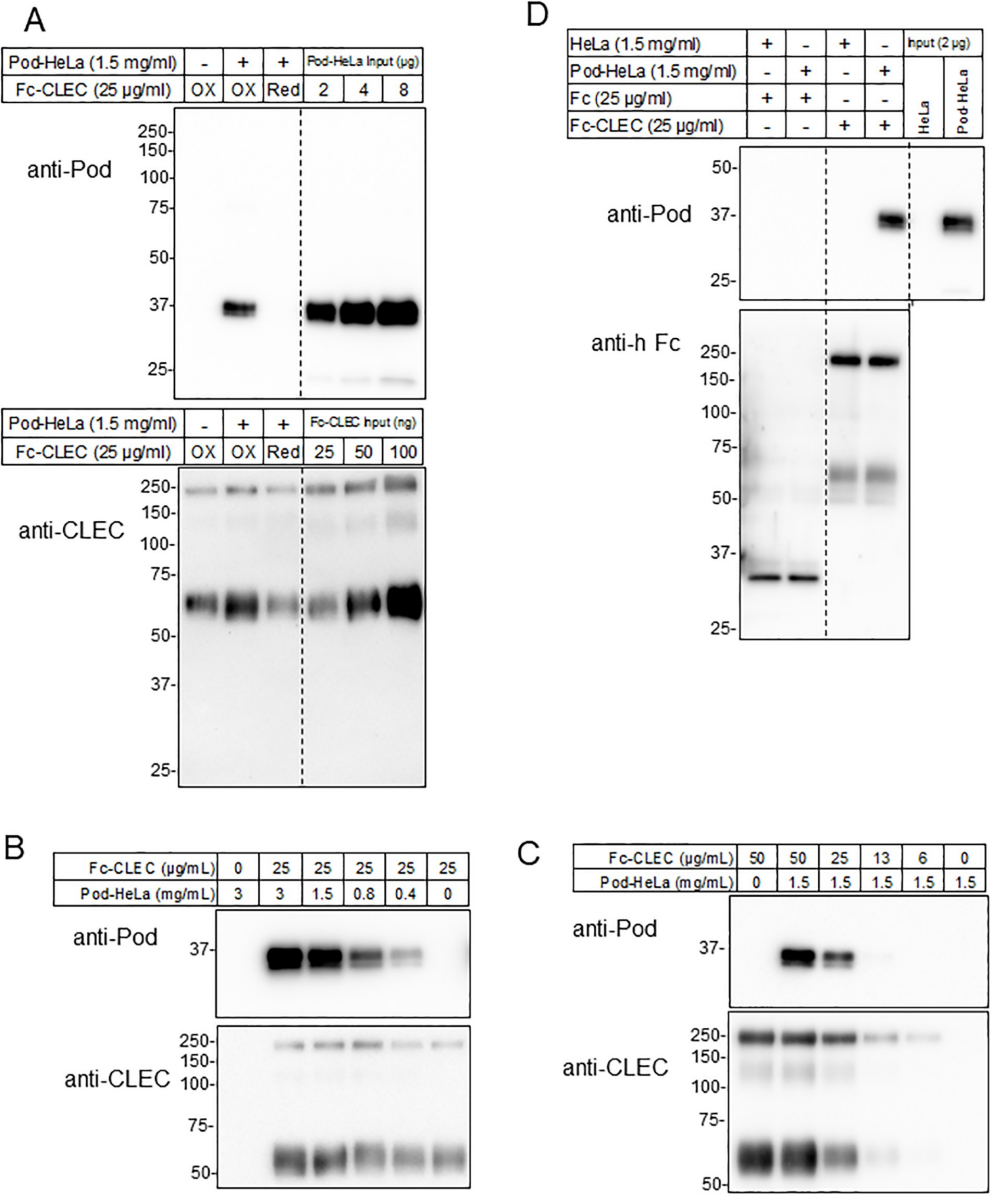
Binding of Fc-CLEC-2 to podoplanin in pod-HeLa cell lysates. (A) Effect of prior reduction of Fc-CLEC-2 disulfide bonds with DTT on its binding to podoplanin. Purified Fc-CLEC-2 in PBS was untreated or treated with 10 mM DTT for 5 h at room temperature. The binding reaction with podoplanin was then performed at final concentrations of 1.5 mg/mL and 25 μg/mL for pod-HeLa lysate and Fc-CLEC-2, respectively. Acid-elution fractions were analyzed by western blotting under reducing conditions using anti-podoplanin (upper blot) or anti-CLEC-2 (lower blot). The indicated amounts of pod-HeLa lysate or Fc-CLEC-2 were also loaded (Input). If the recovery of the pull-down assay is 100%, the maximum amount of podoplanin on the blot corresponds to 8 μg of pod-HeLa lysate and the maximum amount of Fc-CLEC is 65 ng. ‘Ox’ and ‘Red’ represent samples without and with DTT pre-treatment, respectively. (B and C) Western blot analysis of concentration-dependent formation of the podoplanin-Fc-CLEC-2 complex. Complexes were made by mixing the indicated concentrations of non-reduced Fc-CLEC-2 and pod-HeLa lysates. (D) Specificity of the pull-down reaction. Pull down assays were conducted with a combination of lysate from parental HeLa cells or pod-HeLa cells, each at 1.5 mg/mL, and Fc or Fc-CLEC, each at 25 μg/mL, as noted on the above the blots. Acid-elution fractions were analyzed by western blotting under reducing conditions using anti-podoplanin (upper blot) or anti-human Fc-HRP (lower blot). Data shown are a representative result from at least two independent experiments with similar results.

Next, the ability of Fc-CLEC-2 to bind full-length podoplanin from Pod-HeLa cells was evaluated by a pull-down assay, in which we employed a commercially available 0.5 mL centrifugal filter unit when separating bead-bound podoplanin from free podoplanin and other components (Fig 1). To clarify the role of disulfide bonds, Fc-CLEC-2 was reduced with DTT prior to the assay. Although the non-reduced (oxidized) form of Fc-CLEC-2 could robustly sediment 36 kDa full-length podoplanin, DTT-reduced Fc-CLEC-2 could not (Fig 3A upper blot). DTT treatment also partially decreased the recovery of Fc-CLEC-2 (Fig 3A lower blot), possibly due a decreased affinity to Protein A as a results of the cleavage of the disulfide bond connecting two Fc domains. These results suggest that intra- and/or inter-molecular disulfide bond(s) are important for the interaction of CLEC-2 with podoplanin molecules. Under the non-reducing binding conditions, a rough estimation from the input bands in each blot suggested that not more than 20% of podoplanin and almost all of the Fc-CLEC-2 in the reaction mixture were recovered (Fig 3A).

Using the non-reduced form of Fc-CLEC-2, we next sought to determine the optimum concentrations of Fc-CLEC-2 and Pod-HeLa lysates in the pull-down assay by western blotting. When the Fc-CLEC-2 concentration was held constant at 25 μg/mL, the amount of podoplanin sedimented increased with increasing lysate concentrations up to 3 mg/mL (Fig 3B). Similarly, when the concentration of the Pod-HeLa lysate was held constant at 1.5 mg/mL, the amount of podoplanin sedimented increased with increasing Fc-CLEC-2 concentrations (Fig 3C). Without podoplanin or Fc-CLEC-2, no podoplanin was detected in either blot. Thus, the combination of 1.5 mg/mL Pod-HeLa lysate and 25 μg/mL Fc-CLEC-2 was determined to be non-saturated concentrations of each protein and these conditions were used for subsequent experiments (“standard assay combination”).

To confirm the specificity of the pull down, Fc-CLEC-2 was replaced with Fc and Pod-HeLa lysate with parental HeLa lysate in the standard assay combination. Podoplanin was not sedimented by Fc or from HeLa cell lysate (Fig 3D). These results demonstrate the specificity of podoplanin pull down with Fc-CLEC-2.

### Optimum conditions for podoplanin and Fc-CLCE-2 interaction for slot blot analysis

**Fig 4:**
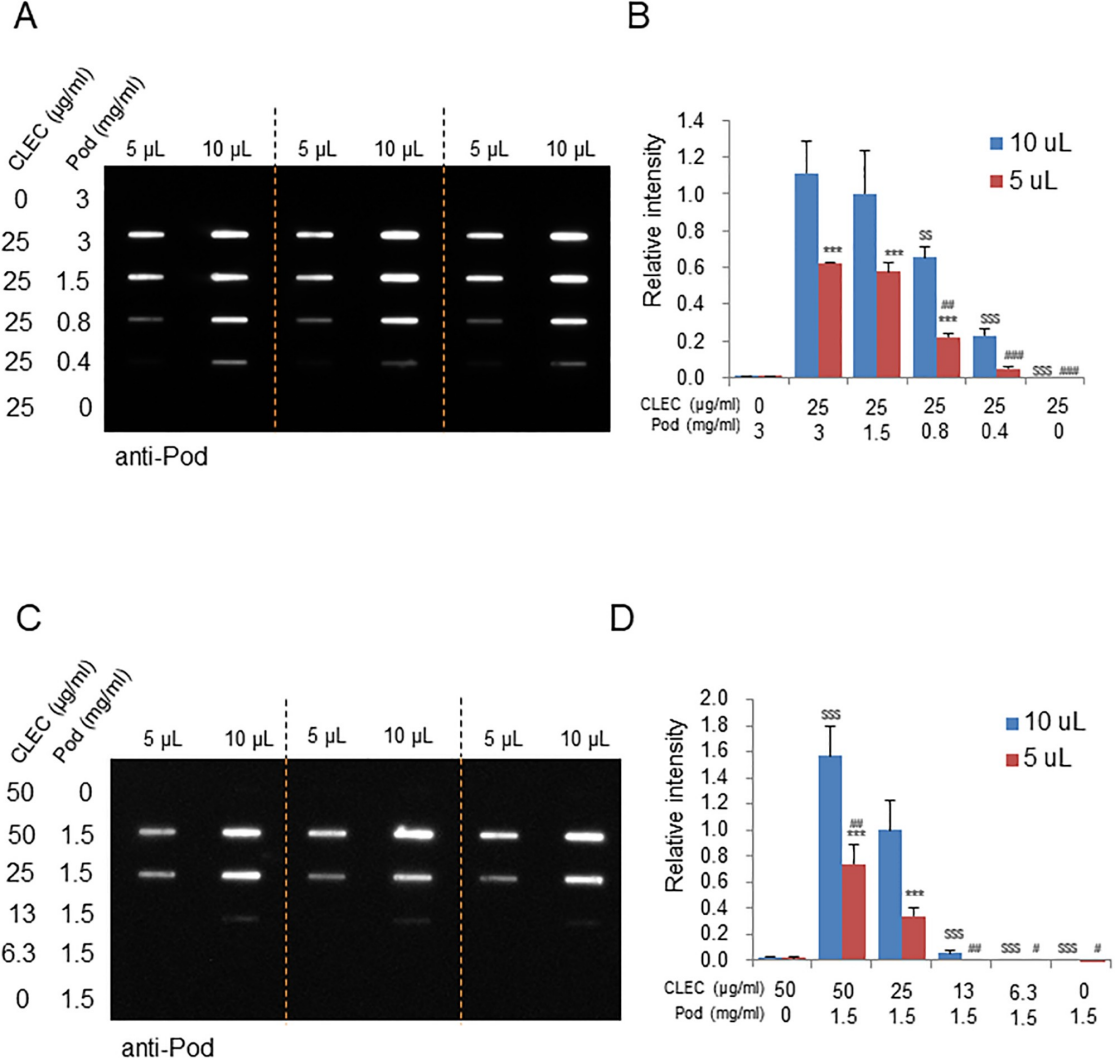
Slot blot analysis of the concentration dependence of podoplanin sedimentation by Fc-CLEC-2 from pod-HeLa cell lysates. (A and C) Slot blot detection of podoplanin-Fc-CLEC-2 complex formation. Acid-elution fractions were obtained from complexes formed with the indicated concentrations of Fc-CLEC-2 and pod-HeLa lysates, as in Fig 3. Either 5 μL or 10 μL of acid-eluted fractions were diluted with PBS to 200 μL, slot-blotted onto PVDF membranes, and stained with an anti-podoplanin antibody. (B and D) Densitometric analysis of the slot bands. The intensity of each slot was normalized to the value obtained at 10 μL loading, from a reaction of 25 μg/mL Fc-CLEC-2 and 1.5 mg/mL pod-HeLa lysate (‘standard combination’). Weak slot signal detected from Fc-CLEC-2 (50 μg/mL) alone (A and B) was due to the cross-reaction of secondary antibody (anti-Rat IgG) with the Fc portion (human sequence) of Fc-CLEC-2. Experiments were performed in triplicate, and the data on the bar chart are expressed as mean ± SD. The statistical significances are noted as follows: ***, P < 0.001 with respect to 10 μL blot in each respective Fc-CLEC and pod-HeLa lysate combination; $ $, P < 0.01, $ $ $, P < 0.001, with respect to the ‘standard combination’ for 10 μL loading series; #, P < 0.01, ###, P < 0.001, with respect to the ‘standard combination’ for 5 μL loading series. Data shown are a representative result from at least two independent experiments with similar results.

Because the acid-elution fraction from the protein A beads contains only Fc-CLEC-2 and podoplanin molecules in principle, we assumed it unnecessary to separate the two proteins on SDS-PAGE before immunodetection (see Fig 3A). Therefore, the acid-elution fractions were directly blotted onto the membrane (Fig 4). To this end, we first assessed the optimum loading volume of the acid-elution fractions obtained from the standard assay combination (1.5 mg/mL Pod-HeLa lysate and 25 μg/mL Fc-CLEC-2). As long as the chemiluminescence signal did not reach saturation, for either podoplanin or CLEC-2 detection, the relationship of loading volume and slot intensity showed a saturation curve with a half maximum loading volume of approximately 5 μL, which contains a maximum of 40 ng of Fc-CLEC-2 per slot (S2A and S2B Fig). Therefore, at a slot loading volume of 5 μL and 10 μL, the relationship between the other reaction combinations and their slot intensity was measured. When the Fc-CLEC-2 concentration was held constant at 25 μg/mL, slot intensity increased in a podoplanin concentration-dependent manner up to 1.5 mg/mL (Fig 4A and 4B). Similarly, when the concentration of the Pod-HeLa lysate was held constant at 1.5 mg/mL, slot intensity increased with increasing Fc-CLEC-2 concentration (Fig 4C and 4D). Without podoplanin or Fc-CLEC-2 in the reaction mixture, essentially no podoplanin slot was detected in either blot. It is of note that for any reaction combinations other than standard combination, a loading volume-dependent increase in slot intensity was also observed (see 5 μL vs. 10 μL). The slot intensity dependency on Pod-HeLa lysate and Fc-CLEC-2 concentrations was essentially identical to the results of the western blot detection of podoplanin, thus verifying the assumption that electrophoresis separation is dispensable for immunodetection of podoplanin in our pull-down assay.

### Validation of the assay system

**Fig 5:**
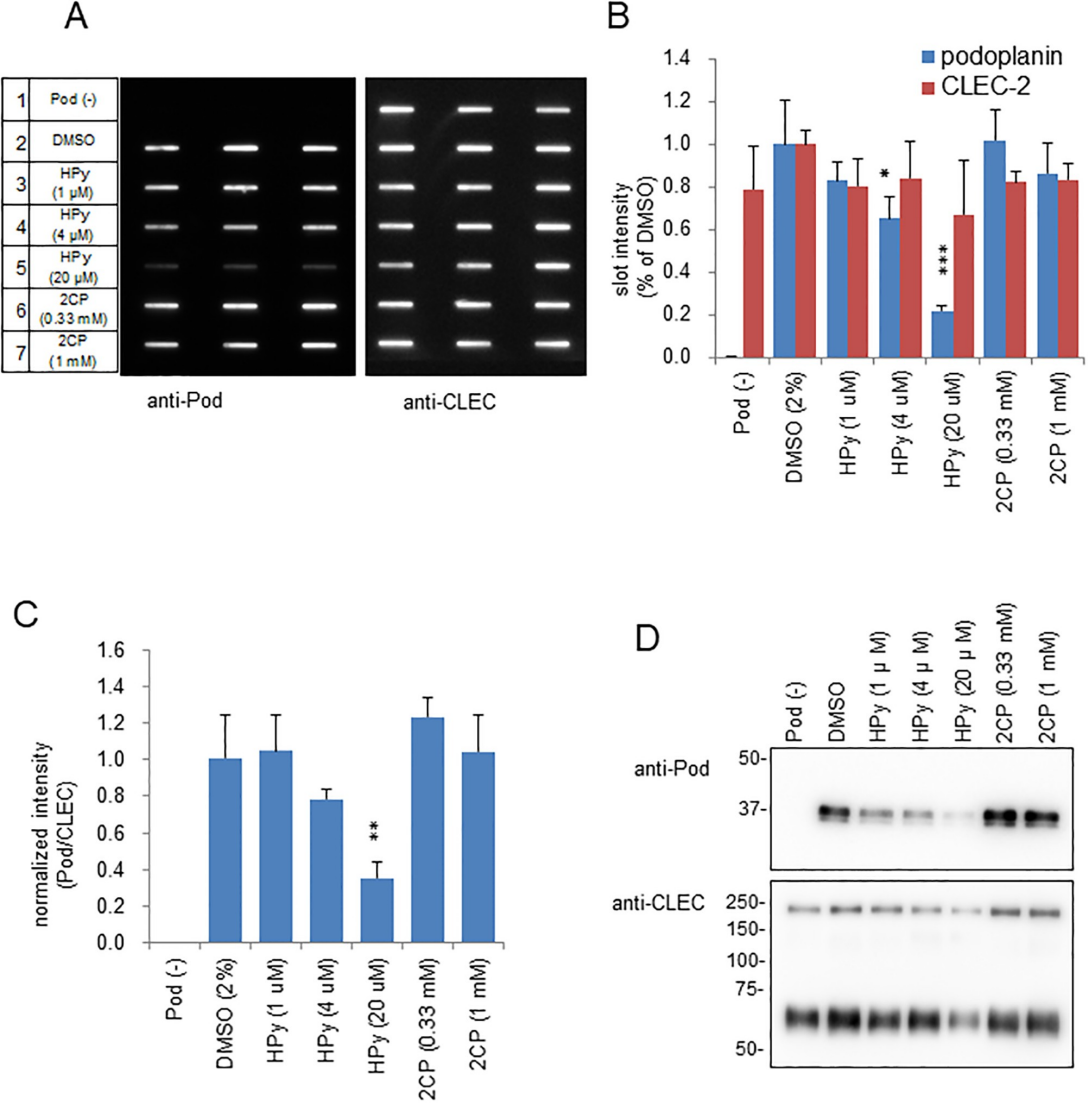
Slot blot analysis of the effects of hematoporphyrin and 2CP on the binding of Fc-CLEC-2 to podoplanin. (A) Slot blot analysis of the levels of podoplanin and CLEC-2 in the elution fraction from complexes formed in the presence or absence of the indicated concentrations of hematoporphyrin (HPy) or 2CP. Complexes were formed with 25 μg/mL Fc-CLEC-2 and 1.5 mg/mL pod-HeLa lysates (standard combination). HPy or 2CP was pre-incubated with Fc-CLEC-2 for 30 min before mixing with pod-HeLa lysates, and the subsequent procedures were conducted under dark. Ten μL of acid-elution fraction were analyzed by slot blotting. The set of blots shown are one representative result performed in triplicate. (B) Relative slot intensities of podoplanin and CLEC-2 in (A). Slot intensities were normalized to those of the DMSO control (2%) and expressed as mean ± SD of triplicate assays. A slight decrease in the recovery of Fc-CLEC in Pod (-) samples was due to its absorption into the plastic ware, which was otherwise prevented by a large amount of cell lysate proteins. (C) The ratio of podoplanin to CLEC-2. Podoplanin slot intensities were normalized to the respective CLEC-2 intensity values in (B). (D) Western blot analysis of acid-eluted fractions. Equal proportion of three replicates of acid-eluted fractions in (A) were pooled, and 10 μL was analyzed by the respective antibody. Data shown are a representative result from at least two independent experiments with similar results. Statistical significance is as follows: *, P < 0.05 and **, P < 0.01, ***, P < 0.001.

To test whether our screening system could indeed select compounds that are capable of blocking the podoplanin-CLEC-2 interaction, 2CP and hematoporphyrin (HPy) were assayed under the condition of standard combination and 10 μL loading. 2CP is a derivative of the anti-platelet agent 4-O-benzoyl-3-methoxy-beta-nitrostyrene (BMNS) and moderately binds CLEC-2 [10]. Hematoporphyrin and its cobalt-binding complex are also reported to bind to CLEC-2 [11]. In our slot blot analysis, however, no inhibitory effect was observed for 2CP even at 1 mM (Fig 5A and 5B). On the other hand, hematoporphyrin above 4 μM had an inhibitory effect. Because hematoporphyrin at high concentrations showed tendency to decrease slot intensity for Fc-CLEC-2, we normalized podoplanin levels according to the corresponding CLEC-2 levels (Fig 5C), which gave an estimate of 70% inhibition by this compound at 20 μM.

To further confirm the inhibitory effect of hematoporphyrin, acid-eluted proteins were analyzed by western blotting (Fig 5D). Consistent with the slot blot results, hematoporphyrin at 20 μM potently decreased podoplanin levels, without significantly affecting CLEC-2 levels. These results verify the effectiveness of our slot blot method and demonstrate that the parallel execution of slot blotting for CLEC-2 can distinguish the target of the test compound, that is, podoplanin-CLEC-2 binding or Fc-protein A binding.

As we noticed that higher concentrations of hematoporphyrin severely decreased Fc-CLEC-2 recovery, we assessed the effect of hematoporphyrin on the interaction between protein A and Fc. As shown in S3 Fig, hematoporphyrin (> 100 μM) significantly inhibited the interaction between protein A and Fc-CLEC-2 as well as between protein A and the Fc moiety at high concentrations. Hematoporphyrin also induced 2-ME-resistant dimerization of the Fc moieties through an unknown mechanism.

## Discussion

Despite the growing attention given to the podoplanin-CLEC-2 interaction as a therapeutic target, reports on inhibitory compounds or screening systems is limited. In this study we have developed a slot blot-based pull down assay for the selection of inhibitor compounds of podoplanin-CLEC-2 interaction. To our knowledge, this is the first report regarding a pull-down based compound screening system utilizing centrifugal filter units and slot blot for increased throughput.

As the binding affinity of CLEC-2 for podoplanin (K_d_ = 25 μM [14]) is not as high as the affinity for a general antigen-antibody interaction (K_d_ = nM ~ pM range), dissociation of the complex is expected to occur if the complex is kept under non-equilibrium conditions, such as during washing steps. To minimize these effects, we employed a commercially available 0.5 mL centrifugal filter unit when separating bead-bound podoplanin from free podoplanin and other components. Although it costs $3 per unit, the use of the centrifugal filter units allowed rapid separation of bound and unbound podoplanin molecules for multiple samples simultaneously, thus minimizing possible intra-assay deviation.

We also employed slot blotting for increased turnaround time and throughput. Because the acid-elution fraction from the protein A beads contains only Fc-CLEC-2 and podoplanin molecules in principle, we assumed it unnecessary to separate the two proteins on SDS-PAGE before immunodetection. We verified this assumption in this study. Furthermore, if one SDS-PAGE gel can ran 12 samples, our 48-well slot blot system can analyze 4 gel equivalents of western blot samples in a single membrane immunoassay. Thus, one run of our assay can analyze 48 samples within a single day.

We conducted a validation of our assay system using two reported inhibitory compounds of podoplanin-CLEC-2 interaction, hematoporphyrin and 2CP. The results demonstrated that while hematoporphyrin was effective, 2CP was not. Our hypothesis is that the different outcomes were due to the difference in their affinity towards CLEC-2. The K_d_ value of 2CP to CLEC-2 is 33 μM [10] whereas that of Co-bound hematoporphyrin is estimated to be between 10–100 pM [11]. Although Co-free hematoporphyrin was used in our assay and its K_d_ value is not available, it can inhibit podoplanin-CLEC-2 interaction to a similar extent as Co-bound forms in an ELISA-based binding assay [11]. Although hematoporphyrin has high affinity, a concentration of 20 μM was required in order for hematoporphyrin to demonstrate an inhibitory effect in our assay system. We concluded that a certain level of affinity (K_d_ << 33 μM) is essential in order to be selected by our assay system. Increasing the sensitivity to levels high enough to enable the detection of the inhibitory effect of 2CP in our assay will be a challenge for us in the future.

An ELISA-based binding assay utilizing immobilized Fc fusion proteins of CLEC-2 and biotinylated Fc fusion proteins of podoplanin has been developed [7] and using a similar ELISA system, Co-hematoporphyrin was successfully selected from a 10,000-compound library [11]. Although these ELISA are superior to our slot blot assay system in terms of throughput and turnaround time, our assay has several potential advantages over ELISA methods. Firstly, in ELISAs, absorption of the Fc-fusion protein onto a plastic surface is inevitably associated with a change in the protein’s structure [17], whereas our solution-phase binding assay has no such structural constraints during the binding reaction between podoplanin and CLEC-2 or the test compound. Moreover, there is no need to covalently attach a biotin moiety, which also affects the protein structure. Secondly, our assay can use cell lysates from HeLa cells stably overexpressing podoplanin, without any prior purification, which enables us to produce a large amount of podoplanin. Thirdly, ELISAs are vulnerable to test compounds with a hydrophobic nature, which may cause dissociation of immobilized Fc-fusion proteins from the matrix (Vroman effect [17]), resulting in a false-positive signal. In our assay, by contrast, test compounds, as well as unbound podoplanin, are washed away before blotting onto the PVDF membrane. All of these potential advantages could contribute to the identification of distinct compounds that cannot be identified with an ELISA-based system.

Besides tumor metastasis, recent studies have demonstrated that the podoplanin-CLEC-2 interaction is important for the development of deep vein thrombosis after mechanical injury [18]. Thus, the need for compounds that inhibit the podoplanin-CLEC-2 interaction is ever increasing and our screening assay system could meet this increasing demand. Furthermore, the methodology established here will be applicable to other protein-protein interaction inhibitor screening systems.

In conclusion, we have established a pull-down and slot blot-based system for the efficient screening of compounds that inhibit the podoplanin-CLEC-2 interaction for use in the treatment of cancer and thrombosis. Our methodology is also applicable to the screening of drugs for other protein-protein interactions.

## Supporting information

S1 Fig Podoplanin expression level in HEK293 cells and purity of Fc. (A) Western blot analysis of podoplanin expression levels in HEK293 cells. As mentioned in Fig 2, Triton X-100-soluble fractions from HEK293 cells and TE11 cell cells were analyzed for podoplanin expression. (B) SDS-PAGE and Coomassie blue staining analysis of Fc under reducing and non-reducing conditions with 2-mercaptoethanol (2ME). Red arrows indicate major bands.
(TIF)

S2 Fig Relationship between loading volume of acid-elution fraction and slot intensity. Acid-elution fraction was prepared and pooled from 10 replicates of the standard assay combination (25 μg/mL Fc-CLEC-2 and 1.5 mg/mL Pod-HeLa lysate). The indicated sample volume was diluted to 200 μL with PBS and slot blotted in duplicate for podoplanin (A) or CLEC-2 (C) as conducted in Fig 4. Slot intensity was plotted against the volume of acid-elution fraction loaded per slot (B and D). Values are shown as a mean ± range of duplicate slots. Data shown are a representative result from at least two independent experiments with similar results.
(TIF)

S3 Fig Effect of hematoporphyrin on the interaction of protein A and Fc domain. Pull down assay was conducted in the presence of the indicated concentration of hematoporphyrin in the same conditions as those mentioned in Fig 5 but included 1.5 mg/mL BSA instead of pod-HeLa lysate. Acid elution fraction was analyzed by western blot using anti-human Fc antibody. Data shown are a representative result from at least two independent experiments with similar results.
(TIF)
