# Supplementary figures and images for: A pull-down and slot blot-based screening system for inhibitor compounds of the podoplanin-CLEC-2 interaction
Source: PLoS One. 2019 Sep 25;14(9):e0222331. doi: 10.1371/journal.pone.0222331 (PMC6760769; doi:10.1371/journal.pone.0222331)

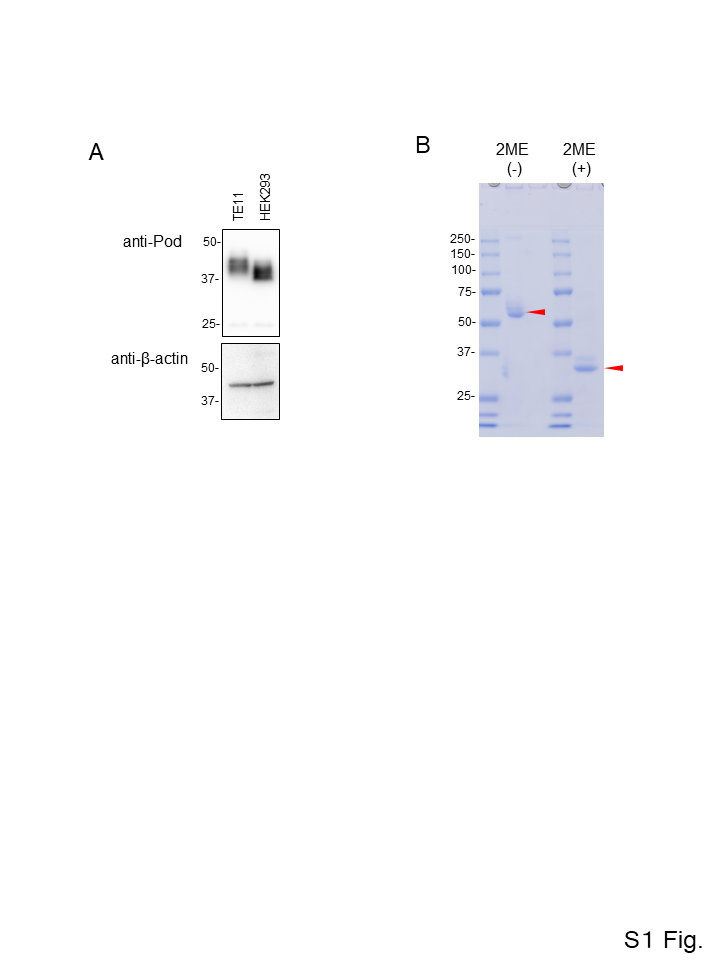

Supplement: S1 Fig — (A) Western blot analysis of podoplanin expression levels in HEK293 cells. As mentioned in Fig 2, Triton X-100-soluble fractions from HEK293 cells and TE11 cell cells were analyzed for podoplanin expression. (B) SDS-PAGE and Coomassie blue staining analysis of Fc under reducing and non-reducing conditions with 2-mercaptoethanol (2ME). Red arrows indicate major bands. (TIF) [file pone.0222331.s001.tif]

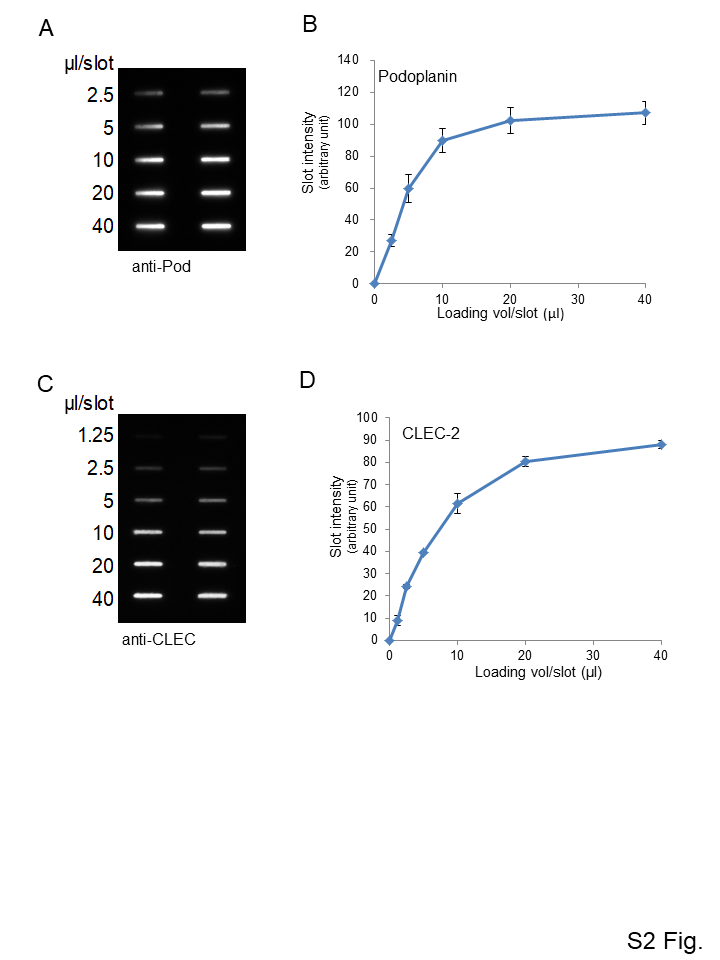

Supplement: S2 Fig — Acid-elution fraction was prepared and pooled from 10 replicates of the standard assay combination (25 μg/mL Fc-CLEC-2 and 1.5 mg/mL Pod-HeLa lysate). The indicated sample volume was diluted to 200 μL with PBS and slot blotted in duplicate for podoplanin (A) or CLEC-2 (C) as conducted in Fig 4. Slot intensity was plotted against the volume of acid-elution fraction loaded per slot (B and D). Values are shown as a mean ± range of duplicate slots. Data shown are a representative result from at least two independent experiments with similar results. (TIF) [file pone.0222331.s002.tif]

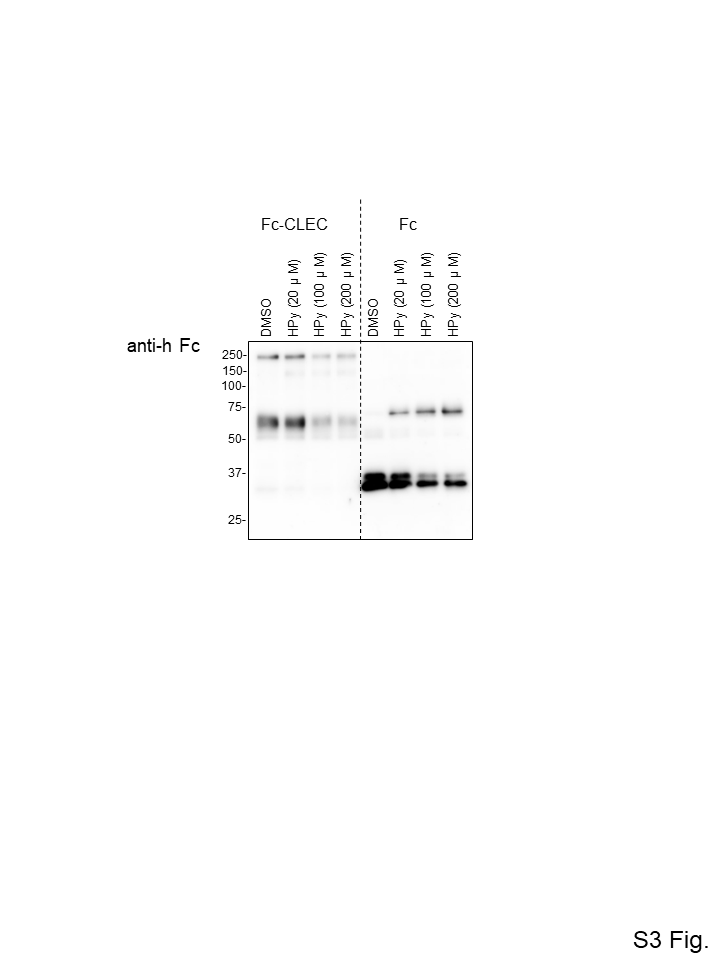

Supplement: S3 Fig — Pull down assay was conducted in the presence of the indicated concentration of hematoporphyrin in the same conditions as those mentioned in Fig 5 but included 1.5 mg/mL BSA instead of pod-HeLa lysate. Acid elution fraction was analyzed by western blot using anti-human Fc antibody. Data shown are a representative result from at least two independent experiments with similar results. (TIF) [file pone.0222331.s003.tif]
